# Otology

**Published:** 1900-12

**Authors:** W. H. Harsant


### OTOLOGY.

One of the principal topics of discussion at the recent International Otological Congress, held in London, was the question of the advisability of **opening the mastoid in chronic suppurative otitis media.**

Professor Politzer, of Vienna, who is undoubtedly the highest authority in all matters relating to ear diseases, opened the discussion.

He laid down nine indications for performing the radical operation:—1. Well-marked caries of the walls of the tympanic cavity. 2. Extensive proliferation of polypi and granulations in the tympanic cavity, growing from the antrum and attic, and recurring even after repeated removal. 3. Carious fistulæ, situated either in the posterior superior wall of the external auditory canal or on the outer surface of the mastoid process. 4. The occurrence of cholesteatoma. 5. A hyperostotic stricture, or complete atresia of the external auditory canal, leading to retention of pus and formation of cheese-like greasy deposits. 6. Facial paresis or paralysis. 7. A painful swelling over the mastoid process, or the formation of an abscess. 8. An obstinate, long-continued, septic, bad-smelling discharge, which has resisted all forms of treatment, especially when the perforation of the drum is situated in the posterior superior quadrant, and the rest of the drum is adherent to the inner wall of the tympanic cavity. 9. Symptoms of tuberculosis appearing in cases of chronic suppuration.

Among the objective symptoms which indicate the necessity for the radical operation should be mentioned fever with a high temperature, preceded by rigor, or fever marked by sudden rising or falling of the temperature, which usually indicates septicæmia. Among subjective symptoms, Professor Politzer considered that persistent or frequently recurring pains in the ear and in the mastoid region, or persistent and fixed pain in the parietal or occipital region, which is increased by percussion, should indicate the necessity of the radical operation.

There would always be cases in which some surgeons, on account of the impossibility of predicting exactly the pathological changes in the temporal bone, will hold the opinion that it is not advisable to await the appearance of well-marked symptoms, and will decide to operate at once; while other surgeons will advocate more conservative methods, from the fact that in numerous cases they have succeeded in healing the discharge without operative interference.

Although he was a strong advocate for the radical operation in suitable cases, he could not agree with those surgeons who not only perform it when the before-mentioned indications are present, but also often for the *mere purpose of curing the discharge.* He thought that in these cases it is not justifiable to have recourse to an operation which, although not necessarily dangerous in the hands of a skilled operator, is still a serious one.

Professor Macewen, of Glasgow, considered that when a pyogenic lesion exists in the middle ear, or its adnexa, which is either not accessible, or which cannot be effectually eradicated through the external ear, the mastoid antrum and cells ought to be opened. He advised the operation even in cases of slight though persistent otorrhœa, which could not be entirely cured by simple means. He considered that in these cases there was always danger of the pyogenic process proceeding inwards, giving rise to symptoms often misunderstood or attributed to other causes, but which might prove fatal or by undermining the constitution might pave the way for the advent of other lesions. After the operation, these patients greatly improved in health, all their old symptoms disappearing along with the cessation of the otorrhœa. Dr. Macewen's personal experiences led him to state that he regarded the operation of opening the mastoid as the safest and most efficient way of eradicating otherwise persistent purulent otitis media. He regarded the operation of opening the mastoid as substantially contributing to the well-being of human comfort and happiness, and as materially lengthening life.

Professor Guye, of Amsterdam, said that one may often be proud of having cured a patient of chronic otorrhœa, without, as well as by operation. He did not find in chronic otorrhœa, without any dangerous symptoms, sufficient indication for the mastoid operation.

Professor Lucae, of Berlin, said that although he was of opinion that the opening of the mastoid is a very important help in the treatment of chronic otorrhœa, yet one may also succeed in many cases without operating. He thought that instead of being proud of saying "I have operated on so many patients," one should be prouder of saying "I have cured so patients without operating."

Dr. McBride, of Edinburgh, desired to associate himself with the more conservative views of Professors Politzer, Guye, and Lucae. He submitted that even if Professor Macewen's proposals were right, they were impracticable because of the frequency of chronic middle ear suppuration. He however, except in special cases, objected to so-called prophylactic operations.

Mr. Cresswell Baber, of Brighton, thought that we were most of us agreed that in chronic suppuration of the middle ear, accompanied by mastoid symptoms, the bone should be opened. The interesting point to consider was whether the mastoid should be opened in cases of chronic suppurative otitis media without any symptoms, except the discharge. In those cases he thought, as a general rule, that first of all every means of arresting the discharge through the meatus (such as careful cleansing, curetting, removal of ossicles, &c.) should be tried, and if the purulent discharge from the tympanum still continued, the risks of pyogenic infection from this focus should be put before the patient or his friends, and the chances of an operation on the mastoid placing him in a safer position explained, though, of course, no certainty of a cure could be promised until the parts had been exposed by operation and the full extent of the disease ascertained.

Many other speakers joined in this discussion, a full report of which will be found in the *Transactions*, edited by Mr. Cresswell Baber, the hon. secretary-general of the congress. The general feeling of the congress seemed to be opposed to the universal operating advised by Dr. Macewen, and to be more in accordance with the conservative views of Professor Politzer. It would be a great pity if it should be widely believed by operating surgeons that it is a good thing to open the mastoid for a chronic purulent discharge from the ear, without any other symptoms, and the recent debate will do much to place this question on a right footing.

W. H. Harsant.

